# C-reactive Protein to Albumin Ratio and Fluid Overload as Predictors of Mortality in Comparison to Pediatric Risk of Mortality (PRISM) III Score in Critically Ill Children in a Tertiary Care Hospital: A Prospective Cohort Study

**DOI:** 10.7759/cureus.102158

**Published:** 2026-01-23

**Authors:** Ankita Mandal, Keerti Swarnkar, Shikha Malik

**Affiliations:** 1 Pediatrics, All India Institute of Medical Sciences, Bhopal, Bhopal, IND

**Keywords:** c-reactive protein (crp) to albumin ratio, fluid overload, multiple organ dysfunction syndrome (mods), pediatric intensive care unit (picu), prism iii score

## Abstract

Objectives: To study the predictive value of the C-reactive protein (CRP) to albumin ratio and fluid overload for the development of Multiple Organ Dysfunction Syndrome (MODS) and mortality in critically ill children, and compare it with the Pediatric Risk of Mortality (PRISM) III score.

Methods: This prospective cohort study was conducted in the pediatric intensive care unit (PICU) of a tertiary care hospital in central India. PRISM-III scoring, CRP, and albumin level were done within 24 hours of admission. Early and cumulative fluid overload were calculated. Sensitivity and specificity were calculated for the CRP-to-albumin ratio and fluid overload for MODS and mortality, and compared with the PRISM III score.

Results: We enrolled 102 critically ill children. The median CRP-to-albumin ratio was significantly higher in non-survivors than survivors (25.30 vs. 3.37, respectively) (p-value <0.001). At a cut-off value of 10.73, it has 72.6% sensitivity and 79.3% specificity. Patients with a ratio of 9.25 or higher were at a significant risk of developing MODS (p <0.0001). CRP-to-albumin ratio is also associated with increased risk for mechanical ventilation and inotrope use. Furthermore, we established a strong positive correlation (coefficient: 0.44; p<0.0001) between the CRP-to-albumin ratio and PRISM III score. Though fluid balance was statistically insignificant for the development of MODS and mortality, it varied significantly between the length of PICU stay (p = 0.007).

Conclusions: CRP to albumin ratio is an inexpensive but statistically significant biomarker having comparable discriminatory power to that of PRISM III score for assessing critically ill children, and fluid imbalance is a significant indicator of duration of PICU stay.

## Introduction

Pediatric intensive care units (PICUs) aim to reduce mortality and morbidity in critically ill children, driving ongoing efforts to predict adverse outcomes for early intervention and resource allocation [1,2]. This study seeks a simple prognostic parameter with sensitivity and specificity comparable to established scores [3], suitable for resource-limited settings like India. While numerous prognostic indicators [4,5] and scoring systems [1,2,6] exist, many are invasive, complex, or lack sufficient sensitivity and specificity, limiting their application. Our focus is on identifying a simple prognostic indicator with comparable performance to established methods.

C-reactive protein (CRP), a pentameric protein produced by the liver, is a positive acute-phase reactant that binds to lysophosphatidylcholine on dead cell surfaces, activating the complement system via C1q. Albumin, the major plasma protein, maintains colloidal osmotic pressure and is a negative acute-phase reactant, decreasing during inflammation [7]. Their ratio has been studied in predicting poor outcomes in critically ill adults, but evidence in pediatric populations remains limited [8-10].

Fluid management is critical in the PICU, where appropriate resuscitation improves outcomes, but excessive fluid administration can be detrimental [5,11,12]. While fluid balance's impact on outcomes is established in specific clinical contexts, evidence specific to early and acquired fluid overload in PICU and its association with adverse outcomes in critically ill children is lacking. Hence, this study aims to assess the predictive value of the CRP-to-albumin ratio and fluid overload for Multiple Organ Dysfunction Syndrome (MODS) development and mortality in critically ill children, comparing these with the Pediatric Risk of Mortality (PRISM) III score.

## Materials and methods

This single-center prospective cohort study was conducted in the medical PICU of a tertiary care hospital in central India from March 2021 to September 2022. Ethical approval was obtained (All India Institute of Medical Sciences, Bhopal, with approval number 2020/PG/July/37, dated February 24, 2021), and recruitment included critically ill patients aged >1 month to <18 years, excluding those with congenital heart disease, nephrotic syndrome, chronic liver disease, or any other condition causing hypoalbuminemia, like severe acute malnutrition, protein-losing enteropathy, or malabsorption syndrome. A total of 102 patients were enrolled based on eligibility criteria, calculated using a previous study's effect size (0.28), with a power of 80% and 5% margin of error [8].

After obtaining informed consent, detailed clinical data, vital signs, and anthropometric measurements were collected upon admission. Routine and specific investigations were performed as clinically indicated. PRISM-III score was assessed. Within 24 hours of admission, CRP and albumin levels were measured. Approximately 4 mL of venous blood was collected in serum-separating vacutainers. After serum separation, estimation of albumin and CRP was done using bromocresol green by auto-analyzer and the technique of turbidometry, respectively. CRP-to-albumin ratio was calculated as $\frac{CRP\left(mg/L\right)}{Albumin\left(1g/dL=10^{-4}mg/L\right)}$.

All patients received treatment according to standard PICU care protocols, including fluid management, and were followed until discharge or death. Fluid balance was meticulously monitored, with intake including intravenous fluids, medications, blood/blood products, and nutritional support, and output measured from urine, stools, drainage, and aspirations such as from ascitic or pleural taps or chest tubes.

Fluid overload was calculated as $\frac{fluid \space intake\left(L\right) - fluid \space output\left(L\right)}{body \space weight\left(kg\right) \space PICU \space admission} \times 100$, with early (≥5% of admission body weight within 24 hours) and cumulative (≥15% during the 14-day study period) measures used [13,14]. Outcome measures included MODS incidence, mortality, PICU stay duration, and requirements for inotropes and mechanical ventilation. MODS was defined as reversible physiological dysfunction involving ≥2 organ systems not primarily involved in the condition leading to ICU admission [15].

Data were tabulated using Microsoft Excel 2019 (Microsoft Corp., Redmond, WA, USA) and analyzed in R software (R Foundation for Statistical Computing, Vienna, Austria, https://www.R-project.org/). Categorical variables were summarized with frequencies, and continuous variables as mean (standard deviation) or median (IQR) based on distribution. Fisher’s exact or Pearson’s Chi-squared tests compared categorical data, while t-tests or ANOVA (parametric) and Mann-Whitney U or Kruskal-Wallis tests (non-parametric) compared continuous variables.

Receiver operating characteristic (ROC) curves and area under the curve (AUC) were used to assess the CRP-to-albumin ratio's predictive performance for MODS and mortality, with sensitivity and specificity calculated using the Youden Index for optimal thresholds. P-values < 0.05 were considered statistically significant.

## Results

A total of 102 patients were enrolled, out of which 73 patients were discharged, and 29 died. Fifty-five (53.92%) were male, and 47 (46.07%) were female patients. The proportion of male patients and median age in the discharged and death groups were not statistically different (p = 0.3 and 0.2, respectively). Out of the total majority of the cases, 24 (23.53%) were neurological, followed by 16 (15.69%) oncology cases, 15 (14.72%) with respiratory illnesses, 12 (11.7%) infectious disease cases, nine (8.82%) immunology cases, and seven (6.86%) hematology cases. Cases, such as rheumatology, gastrointestinal, cardiovascular system, endocrine system, accounted for 3.92% each, and metabolic disorders were 0.98%.

There was no significant difference in the proportion of systems involved in patients who succumbed to death versus patients who were discharged. For death, the major contribution was from oncology cases, whereas neurology cases contributed largely in the case of discharge (Table 1).

**Table 1:** Characteristics of the patients by outcome

Characteristic	Death, N=29	Discharged, N = 73	p-value	Test name and statistics
Age (years)				
Median (IQR)	11.00 (5.00- 14.00)	7.00 (4.00- 11.00)	0.2	Mann-Whitney U = 884.5, Z = -1.20
Range	1.00-16.00	1.00-17.00		
Sex				
Female	11 (37.93%)	36 (49.32%)	0.3	Chi-square test X^2 ^= 0.722
Male	18 (62.07%)	37 (50.68%)		
Primary system involved				
Cardiovascular	2 (6.90%)	2 (2.74%)	0.08	Chi-square test X^2 ^= 15.35
Endocrine	2 (6.90%)	2 (2.74%)		
Gastrointestinal	0 (0.00%)	3 (4.11%)		
Hematology	0 (0.00%)	7 (9.59%)		
Immunology	1 (3.45%)	8 (10.96%)		
Infectious	6 (20.69%)	6 (8.22%)		
Neurology	4 (13.79%)	20 (27.40%)		
Oncology	7 (24.14%)	9 (12.33%)		
Respiratory	3 (10.34%)	12 (16.44%)		
Others (rheumatology, genetic, skin, metabolic, etc.)	4 (13.79%)	4 (5.48%)		

PRISM-III score of the patients was reported to be 3.00 (0.00-5.00) in the discharge group and 9.00 (5.00-13.00) in the death group. This difference was statistically significant (p<0.001). The C-reactive protein and albumin ratio was reported to be 3.37 (1.07-13.01) in the discharge group as compared to 25.30 (11.60-68.02) in the death group, which was also statistically significant, p <0.001. Cumulative and early fluid balance were not found to vary significantly between the two groups (p=0.11 and 0.3, respectively) (Table 2).

**Table 2:** Outcome parameters of the patients as per mortality and MODS IQR: interquartile range, PRISM: Pediatric Risk of Mortality, MODS: Multiple Organ Dysfunction Syndrome​

Characteristic	Death (N=29)	Discharged (N=73)	p-value	Test name and statistics	No MODS (N=66)	MODS (N=36)	p-value	Test name and statistics
PRISM III score median (IQR) [3]	9.00 (5.00-13.00)	3.00 (0.00-5.00)	<0.001	Mann-Whitney U = 434, Z = -4.617	3.00 (0.50-5.00)	8.00 (4.75-12.00)	<0.001	Mann-Whitney U = 556, Z = -4.315
Range	0.00-27.00	0.00-15.00			0.00-23.00	0.00-27.00		
CRP-to-albumin ratio (x10^-4) median (IQR)	25.30 (11.60-68.02)	3.37 (1.07-13.01)	<0.001	Mann-Whitney U = 354, Z = -5.179	3.03 (0.98-8.03)	22.95 (11.47-40.83)	<0.001	Mann-Whitney U = 396, Z = -5.429
Range	2.85-106.60	0.05-52.90			0.05-105.90	1.89-106.60		
Cumulative fluid balance median (IQR)	0.07 (0.03-0.15)	0.06 (0.01-0.13)	0.11	Mann-Whitney U = 828, Z = -1.62	0.06 (0.02-0.14)	0.05 (0.01-0.14)	0.7	Mann-Whitney U = 1126, Z = -0.187
Range	-0.05-0.64	-0.14-0.35			-0.14- 0.57	-0.13-0.64		
Early fluid balance median (IQR)	0.02 (0.01-0.06)	0.02 (0.00-0.03)	0.3	Mann-Whitney U = 909, Z = -1.01	0.02 (0.01-0.04)	0.01 (0.00-0.03)	0.10	Mann-Whitney U = 934, Z = -1.562
Range	-0.02-15.00	-0.04-0.08			-0.04-0.08	-0.02-15.00		

The median (IQR) PRISM-III scores of the patients that developed MODS (8.00 (4.75- 12.00)) were significantly (p <0.001) higher as compared to the PRISM-III score of the patients that did not (3.00 (0.50- 5.00)). Similarly, the CRP-to-albumin ratio median (IQR) value 22.95 (11.47- 40.83) was significantly higher (p <0.001) in the group of patients that developed MODS than the median value of the ratio in the group that did not (3.03 (0.98-8.03)) (Table 2).

Length of stay in the PICU was divided into three categories: >48 hours, >48 hours and up to 7 days, and >7 days. Cumulative fluid balance was found to vary significantly between the groups of patients with p = 0.007, the <48 hours group having the lowest median cumulative fluid values at 0.02 (0.01-0.07), as compared to the highest values in the >7 days group at 0.10 (0.01- 0.18).

Ratio of C-reactive protein to albumin is a statistically significant predictor of mortality and MODS. Patients with a ratio of 9.25 (p <0.0001) or higher are at a significant risk of developing MODS, and those with the values of 10.73 or more are at a higher risk of mortality (p<0.0001). Prediction of mortality can be done with 72.6% sensitivity and 79.3% specificity, whereas MODS predictions are 75.7% sensitive and 80.55% specific with the values of the CRP-to-albumin ratio. However, fluid imbalance has no significant predictive value (Table 3).

**Table 3:** Predictive values of CRP (albumin and fluid imbalance) ROC: receiver operating characteristic; AUC: area under the curve; CRP: C-reactive protein; MODS: Multiple Organ Dysfunction Syndrome; Sn: sensitivity; Sp: specificity

Variable	Threshold	Sn (%)	Sp (%)	AUC (ROC) (95% CI)	p-value
CRP: albumin-mortality	10.73	72.6	79.3	0.832 (0.741-0.906)	<0.0001
CRP: albumin-MODS	9.25	75.75	80.55	0.814 (0.747-0.903)	<0.0001
Fluid overload-mortality	0.01	27.3	93.1	0.601 (0.467-0.713)	0.056
Fluid overload-MODS	-0.046	95.46	19.44	0.477 (0.361-0.593)	0.64

A multiple logistic regression analysis identified several significant independent factors contributing to mortality risk. Firstly, the CRP-to-albumin ratio emerged as a critical predictor. Specifically, for every unit increase in the CRP-to-albumin ratio, there was a 6% increase in the risk of mortality. This was quantified with an adjusted odds ratio (aOR) of 1.060, within a 95% confidence interval (CI) of 1.032 to 1.089. The PRISM score also significantly impacted mortality risk, with an adjusted OR of 1.267, indicating more than a one-fold increase in risk. Furthermore, the need for mechanical ventilation and inotropic support was also a significant predictor of mortality (Table 4).

**Table 4:** Multivariable logistic regression analysis for determining independent predictors of mortality PRISM: Pediatric Risk of Mortality; CRP: C-reactive protein

Variables	Adjusted odds ratio (95% CI)	p-value (Wilcoxon rank sum test)
Age	1.057 (0.968-1.153)	0.216
Sex	0.663 (0.275-1.599)	0.361
PRISM score [3]	1.267 (1.113-1.414)	0.001
CRP-to-albumin ratio	1.060 (1.032-1.089)	0.001
Mechanical ventilation	1.07 (1.01-1.35)	0.003
Inotrope use	1.19 (1.02-1.345)	0.004

**Figure 1:**
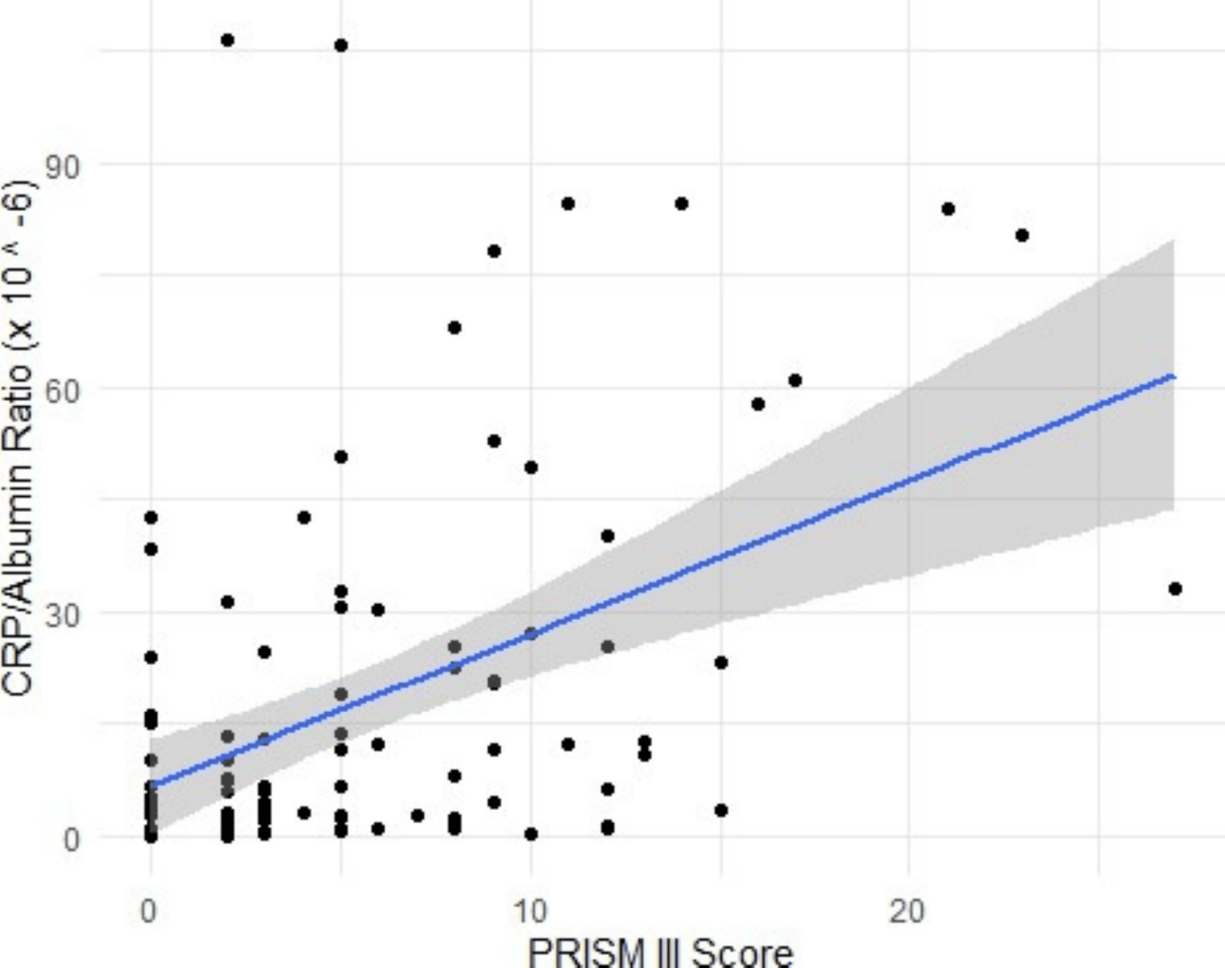
Positive correlation (statistically significant) between PRISM-III and CRP-to-albumin ratio PRISM: Pediatric Risk of Mortality; CRP: C-reactive protein [3]

**Figure 2:**
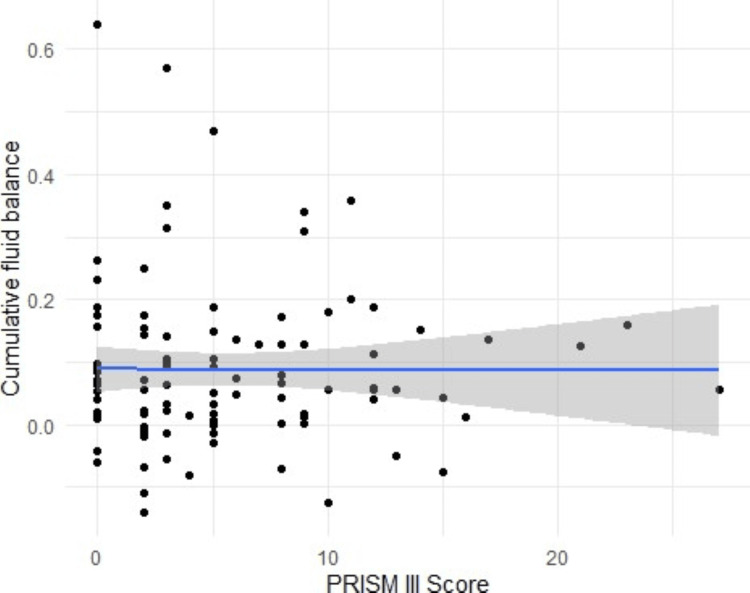
Slight negative correlation (statistically not significant) between cumulative fluid balance and PRISM-III score PRISM: Pediatric Risk of Mortality [3]

PRISM-III and CRP-to-albumin ratio show a positive and significant (p<0.000) correlation. The correlation coefficient is reported to be 0.44 (95% CI: 0.27, 0.58). Although there is a slight negative correlation observed between cumulative fluid balance and PRISM III score (-0.002, 95% CI: -0.196, -0.192), the values are not statistically significant (p = 0.9) (Figures 1, 2).

## Discussion

In the present study, we found that the CRP-to-albumin ratio estimated within 24 hours of admission has significant predictive value (p value <0.001) for MODS and mortality in PICU. These findings are similar to the findings of the study by Mohamed and ElHawary, where they assessed the prognostic value of the CRP-to-albumin ratio in 178 critically ill children [16]. The values reported by them were 18.60 (6.67-32) in the non-survivor group and 4.65 (2.73-11.81) in the survivor group (p <0.001). They also reported PRISM-III values of 7 (3-10) in the non-survivor group and 2 (0-3) in the survivor group. Furthermore, they had predicted a cut-off value of CRP-to-albumin ratio in predicting mortality, the cut-off was ≥25.83 with 41.7% sensitivity and 96.5% specificity, and an AUC of 0.795 (p <0.001). The best cut-off value of the ratio in our study for prediction of mortality was 10.73 and above, with a sensitivity of 72.6% and specificity of 79.3% and AUC of 0.83 (p <0.0001). The cut-off for incidence of MODS was 9.25 with 75.7% sensitivity and 80.5% specificity, and an AUC of 0.814 (p<0.0001). This concluded the significant predictive value of the CRP-to-albumin ratio for MODS and mortality in PICU. The high magnitude of AUC and sensitivity and specificity values of >70% for the cut-offs make the biomarker a suitable candidate for assessment of MODS and mortality in a resource and time-limited facility for accurate and efficient prediction of outcomes such as MODS and mortality.

In our study, for every unit increase in the CRP-to-albumin ratio, there was a 6% increase in the risk of mortality (aOR 1.060, 95% CI 1.032 to 1.089). Mohamed and ElHawary also reported in their study that each unit of increase in the CRP-to-albumin ratio increased the risk of mortality by 1.079 (aOR=1.079) [16]. Similarly, Mohd Amin et al. also concluded that the CRP-to-albumin ratio of ≥ 3 showed significantly higher likelihood for mortality (AUC 0.66 (95% CI 0.54-0.77); p = 0.0150) on day 2 in neonates with necrotizing enterocolitis [10].

In the present study, each unit increase in the PRISM score was associated with a substantial rise in the likelihood of mortality, with an adjusted OR of 1.267. Also, the requirement for mechanical ventilation and inotropic support increased the probability of death by 7%, with an aOR of 1.070, and 19%, aOR of 1.190, respectively. Similar findings were also reported by Mohamed and ElHawary with PRISM-III, mechanical ventilation, and the need for inotropes increased the risk of mortality (aOR=1.27, 0.204, and 0.255, respectively) [16]. Likewise, PRISM score showed adequate discriminatory power for the assessment of prognosis for pediatric patients in a retrospective cohort study conducted by Costa et al. in the PICU of the University of São Paulo Faculty of Medicine Clinics Hospital [17]. Dursun et al. inferred from his study that PRISM III (At a cut-off value of 13, sensitivity and specificity of the PRISM III was 94.4% and 58.3% (AUC: 0.821), respectively) along with need for mechanical ventilation (OR=34.000, P= 0.001, 95%CI 5.272-219.262) and Inotrope support (OR=8.5, P= 0.001, 95%CI 1.318-54.817) were the predictors of mortality [18].

Furthermore, we established a direct correlation between the CRP-to-albumin ratio and PRISM score. Strong positive correlation (coefficient: 0.44; p <0.000) was established between the two parameters, which indicates that as the CRP-to-albumin ratio increases, there is an increase in the PRISM-III score or the severity of illness. Similar findings were noted by Mohamed and ElHawary in their study, where the CRP-to-albumin ratio had almost equivalent discriminatory power to that of the PRISM III score (AUCs 0.795 and 0.793, respectively) [16]. Arslan et al. also compared PRISM IV, PIM 3, and the CRP-to-albumin ratio as prognosticators for patients who had sepsis at the time of admission and found all of them to be reliable predictors of mortality with approximately similar discriminatory power (the AUC for PRISM-4, PIM-3, and CRP-to-albumin ratio were 0.923, 0.896, and 0.795, respectively) [19].

Significant and clinically relevant associations have been established between fluid balance and outcomes of critically ill children in several pathologies, such as acute lung injury, acute kidney injury, severe sepsis, or patients requiring renal replacement therapy [20,21]. However, no such correlation between disease severity and fluid balance was observed in our study. There is a very weak and not statistically significant negative correlation between fluid balance and severity of disease as assessed by PRISM III score.

In the current study, there was no statistically significant difference between the mean fluid balance in survivors and non-survivors or between the groups that did and did not develop MODS. These results are like the study by Kong et al., where they also observed no difference between the survivors and non-survivors' mean fluid balance (p=0.992) [22].

In our study, we observed fluid balance to vary significantly between different durations of PICU stay (p = 0.007). Similar observations were made by Kong et al., where they observed a weak but statistically significant positive correlation between fluid overload and duration of PICU stay (coefficient = 0.148; p = 0.009) [22].

These findings suggest that although fluid overload/balance might not be a clinically significant biomarker in predicting mortality or severity of the disease, it can predict the duration of ICU stay. The higher the value of fluid overload, the longer the PICU stay is expected.

The common practice of fluid resuscitation in critically ill children leads to a host of adverse effects and has been linked to increased mortality [23-25]. In our study, fluid overload has been shown to have direct consequences for the duration of PICU stay, but not other variables such as mortality or MODS. Fluid overload/imbalance has been a controversial subject in assessing critically ill children, and its association with various outcome variables such as mortality, organ dysfunction, and others has been debated rigorously in the literature [26,27].

The strengths of this study were that it is a prospective cohort study, which minimizes recall bias and strengthens causal inference. Clinically relevant, as it evaluates inexpensive, widely available biomarkers (CRP, albumin) useful in resource-limited settings, compares new markers against an established score (PRISM III), providing context. The main limitation of this study was that it was a single-center, small-cohort study, making the generalization of findings difficult, and the PRISM III score was validated on various groups of patients (medical, surgical, trauma). Here, the study population is predominantly medical. Also, albumin was measured using bromocresol green by an auto analyzer, which is prone to overestimation, especially in hypoalbuminemia, compared to bromocresol purple.

## Conclusions

The CRP-to-albumin ratio is an inexpensive, clinically valid, and statistically significant biomarker having comparable discriminatory power to that of the PRISM III score, in assessing critically ill children and for the prediction of mortality and MODS.

Fluid imbalance is a significant indicator of duration of PICU stay, but not mortality and MODS, thus warranting further exploration into the physiology and hemodynamics of pediatric patients. The inconclusive, yet statistically significant associations of fluid imbalance with various outcomes in critically ill children continue to be a subject of assessment, requiring larger cohort studies with significant statistical power.
